# Primary Sphenoidal Sinus Lymphoma with Initial Presentation as Unilateral Abducens Nerve Palsy Symptom

**DOI:** 10.1155/2018/5305963

**Published:** 2018-07-09

**Authors:** Xijing Mao, Lifang Jin, Bochi Zhu, Honghua Cui, Min Yao, Gang Yao

**Affiliations:** ^1^Department of Neurology, The Second Hospital of Jilin University, China; ^2^Department of Hematology and Oncology, The Second Hospital of Jilin University, China; ^3^Department of Pathology, The Second Hospital of Jilin University, China

## Abstract

A 48-year-old man presented with 3 days of mild horizontal diplopia in the left direction, followed by the onset of headache 17 days later. A physical examination revealed isolated left abducens nerve palsy. Head computed tomography (CT) and magnetic resonance imaging (MRI) scans revealed soft-tissue density neoplasms that occupied the sphenoidal sinus and further invaded to destroy the clivus. Immunohistochemical staining of neoplasms was performed from biopsies samples. The pathological diagnosis was extranodal natural killer (NK)/T-cell lymphoma (ENKL), nasal type, associated with Epstein-Barr virus (EBV). The patient subsequently exhibited secondary symptoms (fever, night sweats), enlarged lymph nodes, renal metastases, and hemophagocytic syndrome, with clinical diagnosis stage IV of ENKL. The patient has a poor prognosis. This report is unique in two aspects: the unilateral abducens nerve palsy as the initial and isolated symptom of ENKL, and the primary sphenoidal sinus ENKL.

## 1. Introduction

Abducens nerve palsy is a common clinical finding in neurology practice and the etiology of the palsy is complicated. An accurate diagnosis is usually made through the cooperation of different departments, such as the ophthalmology, otolaryngology, neurology, pediatrics, pathology, and neuroimaging. The common causes of unilateral abducens nerve palsy are neoplasm and vascular disease in middle-aged people [1]. Extranodal natural killer (NK)/T-cell lymphoma (ENKL), nasal type, is the common nasal lymphoma in Asian and South America male adults [2]. The nose and maxillary sinuses are the common initial site of involvement while the sphenoidal sinuses are rarely affected. Multiple cranial nerve deficits or bilateral abducens nerve palsy associated with ENKL have been reported [3], but isolated unilateral abducens palsy is rarely reported. Herein we report a case with unilateral abducens nerve palsy as initial symptom in the primary sphenoidal sinus ENKL and investigated the clinical feature of the diagnosis and therapy.

## 2. Case Report

**Figure 1:**
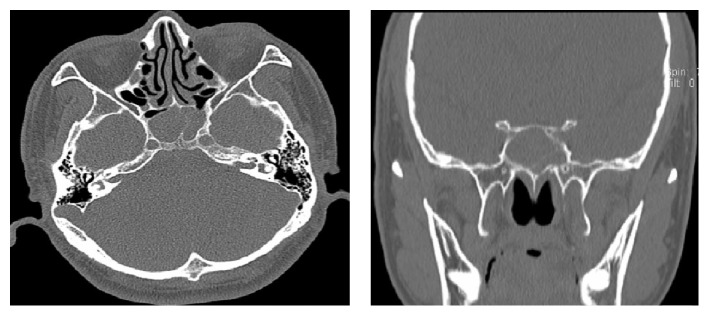
CT scan showed soft-tissue density neoplasms filling with sphenoidal sinus.

**Figure 2:**
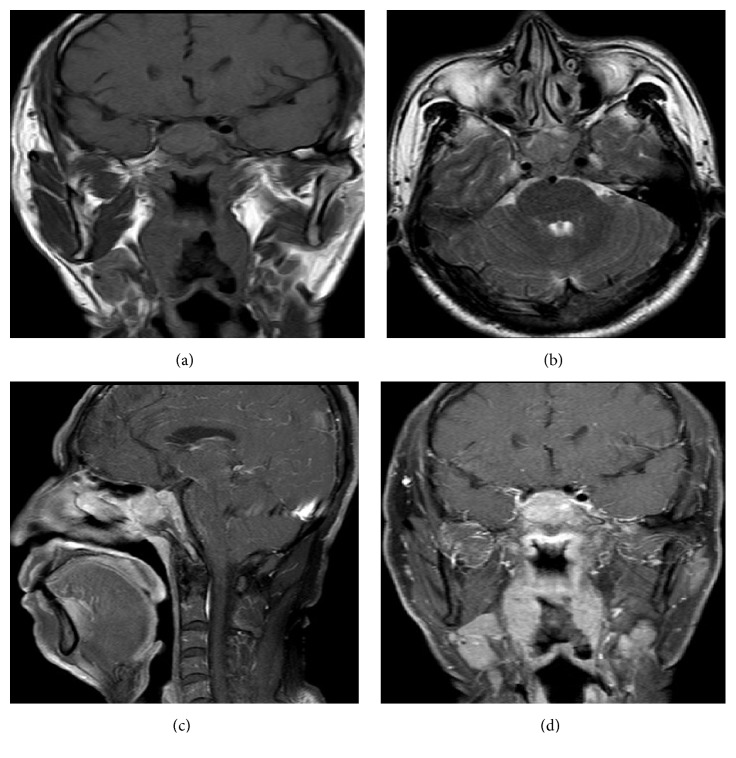
Sagittal T1-weighted magnetic resonance image (MRI) and coronary T2-weighted MRI revealed a mass occupying the sphenoidal sinus (a,b). Gadolinium-enhanced MRI demonstrated the neoplasm with homogenous soft-tissue lesion occupying the sphenoidal sinus and destroying the clivus (c,d).

**Figure 3:**
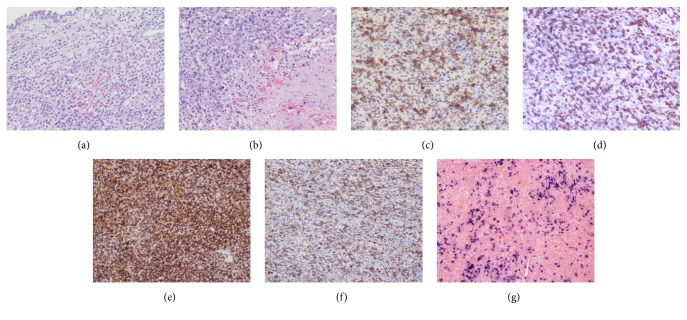
Pathological photomicrographs demonstrated that the mucosa was intact and expanded by a diffuse infiltrate of lymphoma cells (a, H&E). The mucosal lymphoid infiltrate was destructive, resulting in necrosis. The medium-to-large transformed cell nuclei had an irregular nuclear folding with granular appearance (b, H&E). Positive immunohistochemical staining was recorded for (c) CD56, (d) CD3, (e) LCA, (f) CD38, and (g) EBER* in situ* hybridization (original magnification: ×200).

**Figure 4:**
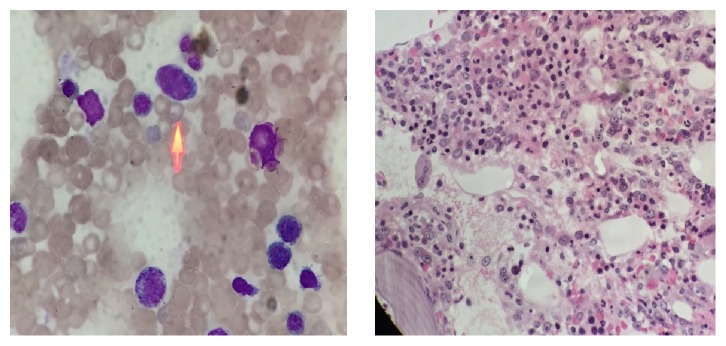
Bone marrow smear and biopsy showed active hyperplasia, immature lymphocytes accounting for 3% of heterotypic large cells with scattered distribution.

A 48-year-old man presented at the ophthalmologic out-patient department with a 3-day mild horizontal diplopia in the left direction followed by the onset of headache 17 days later. He denied nasal obstruction, epistaxis, nasal discharge, pain, hyposmia, and nasal swelling. There was no history of fever, weight loss, or nocturnal sweating. He had no history of diabetes, hypertension, or any neurological disease. On physical examination, cardiopulmonary examination was normal and neither lymphadenopathy nor hepatosplenomegaly was observed. Neuroophthalmologic examination revealed normal visual acuity, fields, and fundi. The pupils were equal and reactive to light and near stimuli. There was no ptosis, but there was limitation of movement of the left eye when he gazed to the left side. Function of the remaining cranial nerves was normal. There were no sensory or motor deficits in the upper and lower extremities; all tendon reflexes were normal. He was found to have isolated left abducens nerve palsy. Computed tomography (CT) scanning revealed soft-tissue density neoplasms filling the sphenoidal sinus (Figure 1). Magnetic resonance imaging (MRI) scanning with gadolinium injection was performed and revealed a homogeneous mass lesion (2.8cm x 2.3cm x 2.9cm) occupying the sphenoidal sinus and invading and destroying the clivus (Figure 2). Rhinoendoscopy revealed a mass at the sphenoidal sinus which was biopsied and histological examination revealed a malignant lymphoma. The immunohistochemical staining of tumor tissues showed CD3^+^, CD56^+^, Ki67>80%, LCA^+^, CD38^+^, and CD20^−^ (Figure 3). The lymphoma cells were positive for EBER* in situ* hybridization. The pathological diagnosis was ENKL. Plasma EBV PCR yielded 1.18 x 10^6^ copies/ml. Ten days later the patient had the B symptom (fever, night sweats). The enlarged lymph nodes were checked in the neck, bilateral subclavian, alar, and inguinal. Contrast enhanced CT showed renal metastases. Bone marrow smear and biopsy showed active hyperplasia, immature lymphocytes accounting for 3%, and heterotypic large cells having a scattered distribution (Figure 4). Flow cytometry analysis showed lymphocytes accounting for 6.8% and suggested phenotypic abnormal NK cells in the bone marrow. Cerebrospinal fluid analysis showed glucose (2.87mmol/L) and protein content (0.22g/L) with normal cell count and no malignant cells. Blood analysis showed complete blood cell reduction. The second bone marrow biopsy suggested hemophagocytic syndrome [4]. The clinical diagnosis was stage IV of ENKL. The patient asked to be transferred to the community hospital.

## 3. Discussion

This report is unique in two aspects: the unilateral abducens nerve palsy as initial and isolated symptom of ENKL, and the primary sphenoidal sinus ENKL.

The abducens nerve exits the pons, runs along the bony clivus, enters the cavernous sinus through Dorello's canal, and subsequently runs through the middle of the cavernous sinus in close relation to the internal carotid artery medial to CN III, IV, and V [5, 6]. The abducens nerve is the most caudally and medially situated nerve in the sinus and is more vulnerable to pathologic lesions that involve the lateral part of the cavernous sinus. When a mass expands from the sphenoidal sinus, as in our patient, it invades the neighboring cavernous sinus and abducens nerve palsy is the common initial symptom. The incidence of cranial nerve palsy in nasopharyngeal neoplasms is 34-39% and most cases present with multiple cranial neuropathies [7, 8], but in our case only the left lateral abducens nerve was involved. Unilateral abducens nerve palsy may be caused by direct brainstem compression, intracisternal involvement, or tumor invasion of the clivus, parasellar structures, and systemic disease such as diabetes mellitus or hypertension pressure. In our case there was no evidence of brainstem and prepontine cistern involvement or compression as evidenced by gadolinium contrast MRI. The mass was confined to the sphenoidal sinus by MRI and rhinoendoscopy, compressing the left cavernous sinus and posteriorly invading the clivus, but not invading anteriorly towards the nasopharynx, so the patient experienced no nasal problem.

Epidemiological data show that the causes of unilateral abducens nerve palsy are closely related to age as trauma and tumors are common in children [9] and neoplasms and ischemia are common in middle-aged people, while high blood pressure or diabetes are common in elderly people [1, 10]. However, idiopathic isolated abducens nerve palsy comprises 26% of all patients with abducens nerve palsy, making the diagnostic plan difficult with abducens nerve palsy without other symptoms [11]. If a patient complains of diplopia caused by difficulty in abducting the muscles, a thorough workup should be performed to find the possible causes so as not to delay treatment.

ENKL is an entity of non-Hodgkin's lymphoma, mostly apparent in the nasal or paranasal area and is characterized by extensive extranodal involvement of NK or T cells [12, 13]. Susceptibility is domicile or ethnicity-related, being more common in Asia and South America than in Western Europe and North America [14]. The incidence of ENKL is 2-10% of the total number of non-Hodgkin's lymphoma, accounting for 90% of the nasal lymphoma in male adults [15]. Our patient is a 42-year-old Chinese man, presenting with initial unilateral abducens nerve palsy without nasal obstruction, nasal bleeding, and the typical progressive nasal facial damage symptoms of ENKL. CT showed soft-tissue density neoplasms filled with sphenoidal sinus, which is hard to differentiate with sphenoid sinusitis and other benign or malignant lesions. Previously reported imaging characteristics of ENKL are nasal cavity mass associated with sinus involvement, mild bone destruction with middle turbinate for CT, an equal or low T1 signal, and slightly higher T2 signals that can be enhanced with mild degree for MRI [16]. These characteristics are consistent with our ENKL case and play an important role in early diagnosis.

The diagnosis of ENKL must be based on pathologic immunohistology. The histopathology of ENKL is characterized by vascular central lesions, where the polymorphous lymphoma cells invade around small blood vessels or vascular tissue, resulting in vascular obstruction and tissue ischemia and extensive necrosis [17]. However, angiocentric growth is not always present and angiocentricity can be observed in other lymphoma types [18]. In our case the pathology revealed a medium-to-large transformed cell infiltrate in blood vessels, resulting in necrosis. These transformed cell nuclei have an irregular nuclear folding with granular appearance. The tumor cells have a CD56^+^CD3^+^ immunophenotype characteristic of NK cells.

The etiology of ENKL is unclear, but as Epstein-barr virus is detected in tumor cells in virtually all cases, ENKL is therefore regarded as an EBV-associated lymphoma [19]. ENKL is not sensitive to chemotherapy because the lymphoma cells can express P-glycoprotein [20] that mediates multidrug resistance. Involved-field radiotherapy followed by chemotherapy is regarded as a standard treatment. ENKL has a poor prognosis, which is usually worse than that associated with lymphomas at other sites in the body [15]. Multivariate analysis revealed that clinical stage, performance status, extranodal involvement, and disease type are significant and independent prognostic factors [21]. In our case the patient had many adverse prognostic factors and deteriorated very quickly following diagnosis.

To conclude, the diagnosis and treatment of unilateral abducens nerve palsy associated with ENKL are often delayed and require integration of ophthalmic, otolaryngological, neurological, and pathological assessments between clinical departments. A thorough workup should be performed including eye, ear, nose, and pharynx inspections. When adult males present with unilateral abducens nerve palsy and nasal sinuses lesions associated with sinus involvement, and imaging features of bone damage are not apparent, clinicians should be highly vigilant to rule out NK/T lymphoma. Confirmatory pathological histology, especially immunohistochemical examination, should be conducted so as to prevent the misdiagnosis of sinusitis. Radiotherapy followed by chemotherapy can improve prognosis.
